# Cone Photoreceptor Sensitivities and Unique Hue Chromatic Responses: Correlation and Causation Imply the Physiological Basis of Unique Hues

**DOI:** 10.1371/journal.pone.0077134

**Published:** 2013-10-21

**Authors:** Ralph W. Pridmore

**Affiliations:** Department of Cognitive Science, Macquarie University, Sydney, New South Wales, Australia; University of British Columbia, Canada

## Abstract

This paper relates major functions at the start and end of the color vision process. The process starts with three cone photoreceptors transducing light into electrical responses. Cone sensitivities were once expected to be Red Green Blue color matching functions (to mix colors) but microspectrometry proved otherwise: they instead peak in yellowish, greenish, and blueish hues. These physiological functions are an enigma, unmatched with any set of psychophysical (behavioral) functions. The end-result of the visual process is color sensation, whose essential percepts are unique (or pure) hues red, yellow, green, blue. Unique hues cannot be described by other hues, but can describe all other hues, e.g., that hue is reddish-blue. They are carried by four opponent chromatic response curves but the literature does not specify whether each curve represents a range of hues or only one hue (a unique) over its wavelength range. Here the latter is demonstrated, confirming that opponent chromatic responses define, and may be termed, unique hue chromatic responses. These psychophysical functions also are an enigma, unmatched with any physiological functions or basis. Here both enigmas are solved by demonstrating the three cone sensitivity curves and the three *spectral* chromatic response curves are almost identical sets (Pearson correlation coefficients *r* from 0.95–1.0) in peak wavelengths, curve shapes, math functions, and curve crossover wavelengths, though previously unrecognized due to presentation of curves in different formats, e.g., log, linear. (Red chromatic response curve is largely nonspectral and thus derives from two cones.) Close correlation combined with deterministic causation implies cones are the physiological basis of unique hues. This match of three physiological and three psychophysical functions is unique in color vision.

## Introduction

The color vision process starts with three cone photoreceptors (Short-, Medium-, Long-wavelength sensitive, or *SML*) transducing light quanta into electrical signals. These lead through the retina and lateral geniculate nucleus (LGN) of the thalamus to cortex and finally color perception, whose essential percepts are the four unique or pure hues [1], red, yellow, green, blue (*rygb*). A unique hue is indivisible. No unique hue contains any part of another: for any observer there is, for example, a certain blue that is not greenish or reddish or yellowish. Two fundamental characteristics of color are that the thousands of discernible hues: (a) can all be mixed by three primary colors broadly named red, green, blue (RGB) for mixing lights (or cyan, magenta, yellow, for mixing pigments); and (b) can all be described in terms of the four unique hues. For example, orange is yellow-red, and purple is red-blue, but no hue name other than red can describe unique red.

These two characteristics, whose relationship is complicated by their different numbers of categories (three and four), underlie the two principal theories of color vision. The first is the Young-Helmholtz or trichromatic theory [2], [3], which correctly predicts three types of cone photoreceptors in the eye, and the minimum number (three) of color mixture primaries. The second theory is Hering's opponent-colors theory [4] which predicts four unique hues and their opponent pairs, yellow-blue and red-green (*y-b*, *r-g*).

**Figure 1:**
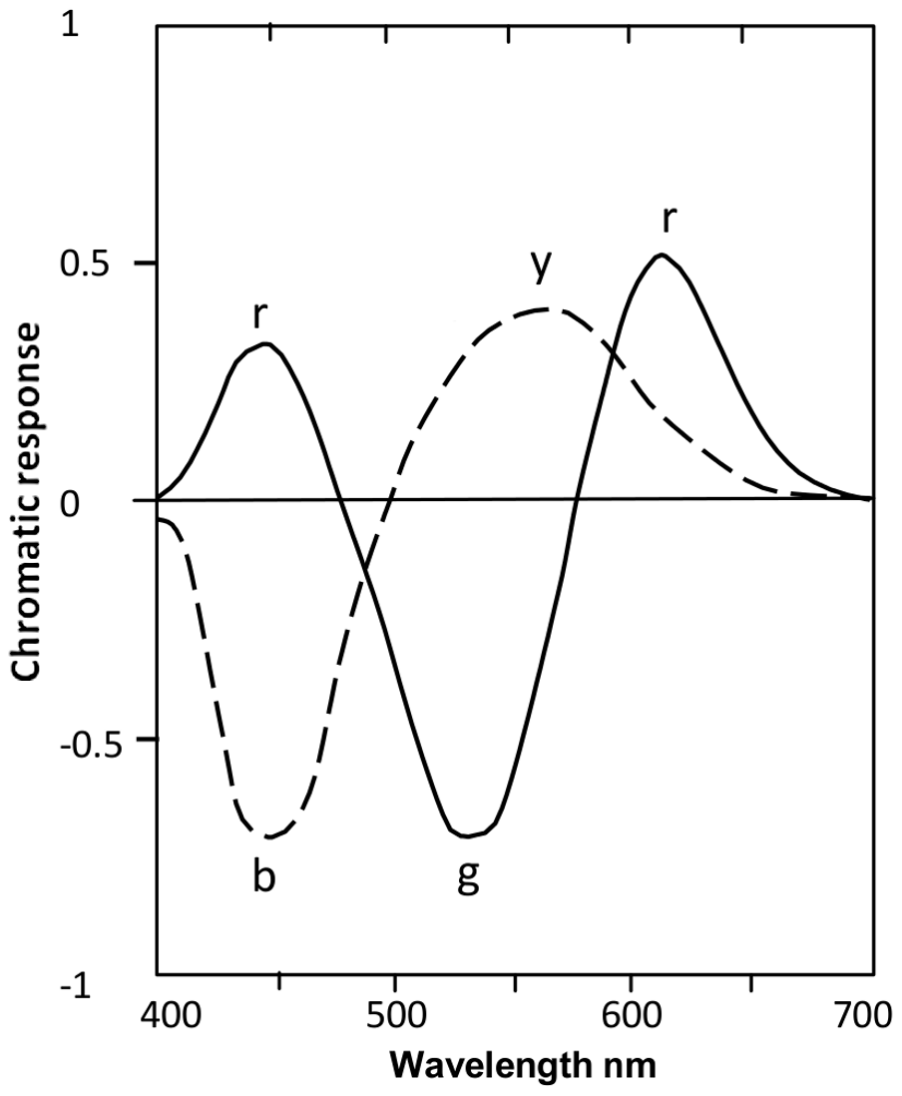
Opponent-color chromatic responses. Solid curve shows *r-g* and dashed curve shows *y-b* response functions, for the spectrum and neutral adaptation. Re-drawn from Hurvich [9].

Both trichromatic and opponent-color theories claim direct relationships with cone responses. Multistage color vision models comprise two main stages: cones, and opponent-color mechanisms, which are theorised to account for color appearance. Each opponent mechanism comprises two chromatic response curves and their two unique hues. See Fig. 1. Opponent-colors theory was first quantified in the Judd-Muller [5] model of color vision and in the first of several Hurvich-Jameson models [6], [7], [8], [9], which led eventually to the so-called Standard Model of the opponent-colors theory of color vision.

Fig. 1 is adapted from the Hurvich 1981 model: consider the *y-b* opponent-color mechanism. When it is in equilibrium (that is, its two chromatic response curves *y* and *b* intersect at the null response level), only one curve, the *g* curve, remains operative (i.e., at a non-zero response level). The equilibrium wavelength marks a unique hue, green in this case. From hue naming experiments [6], [10], [11], discussed further below, observers state each of four unique hues is pure and contains nothing of the other hues.

**Figure 2:**
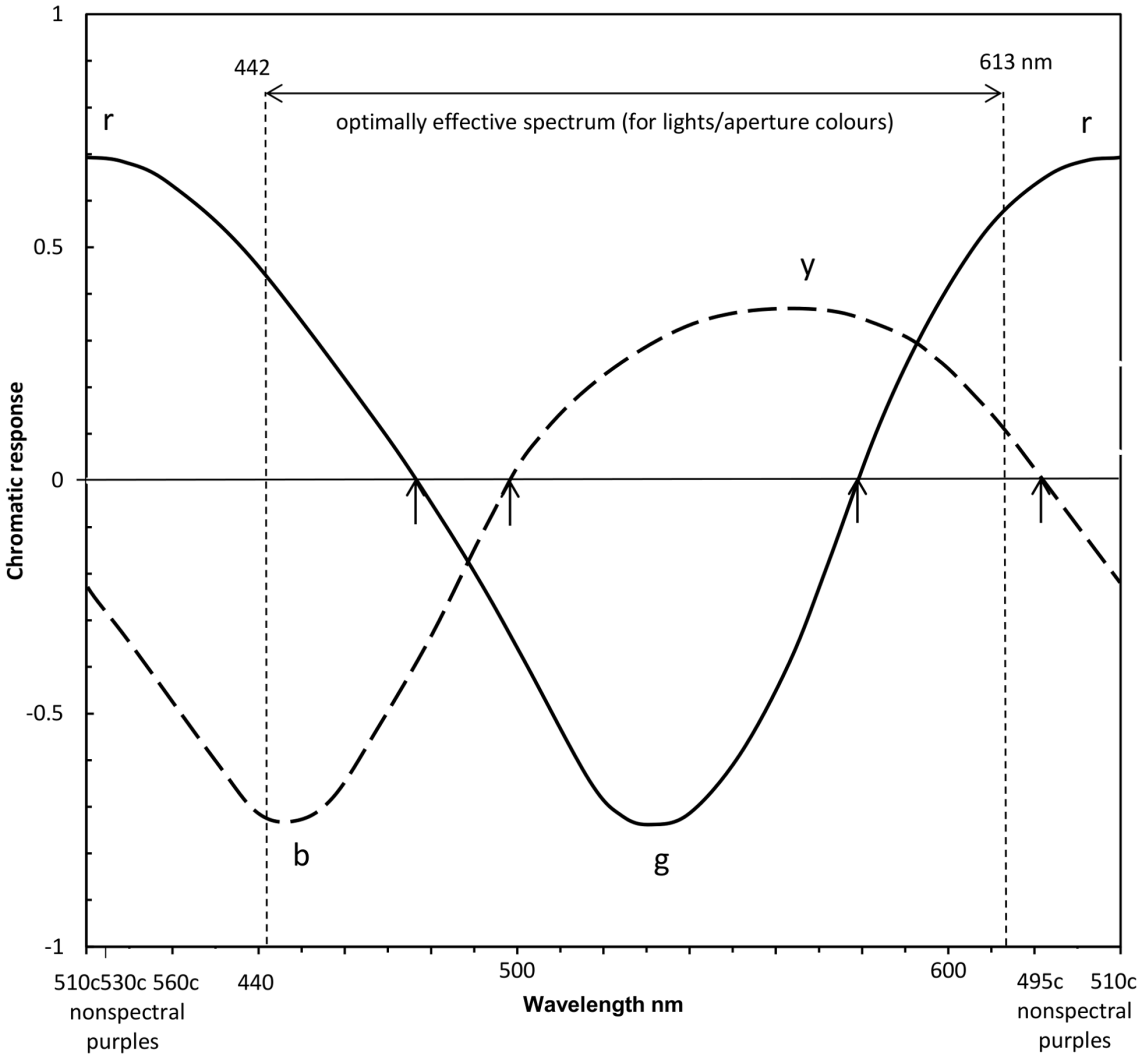
Opponent-color chromatic responses for the hue cycle. As Fig. 1 but expanded from the spectrum to the hue cycle. Vertical dotted lines labeled 442 and 613 nm represent limits to monochromatic optimum color stimuli for lights/aperture colors (and thus limits to the optimally effective spectrum; see text). An arbitrary interval is added for the nonspectral part of the hue cycle (purple and some red hues). Arrows indicate unique hues at 475, 498, 578, and 495 c (i.e., complementary to 495 nm), the latter from other unique hue data on aperture colors [1].

Fig. 1 is spectral so cannot show the *r* curve through the nonspectral region and the location of unique red. So these are shown in Fig. 2 by allowing an arbitrary interval on the x-axis (approximating the metric in [12]) for the nonspectral part of the hue cycle. The nonspectral interval is indicated by complementary wavelengths, e.g., 510 c. The two *r* lobes about 440 and 615 nm have been calculated colorimetrically to form the *r* curve over the nonspectral hues, peaking about 510 c. The spectrum for aperture colors (or lights) is limited to the range 442–613 nm, the limits of monochromatic optimum color stimuli [13], [14]), beyond which the compound colors (nonspectrals), admixed from the optimal wavelength pair 442+613 nm, become the optimal color stimuli. Notably, the 442 and 613 nm pair give colorimetric confirmation of the two spectral peaks for experimental *r* response about 440–445 and 610–615 nm in all hue cancellation data sets. This contrasts with some models, as distinct from hue cancellation experimental data, that omit the short-wavelength lobe to the *r* curve and thus are unable to predict reddish blue (violet) hue at the short wavelength end of the spectrum..

Color vision models derive opponent-color chromatic responses and their unique hues from combinations of cone responses, which vary between vision models. The unique hues' importance has long been known to art [15] and science [4] yet the physiological location or basis of the unique hues remains unknown and controversial [16], [17], [18], [19], [20], [21], with suggested locations varying from retina to LGN and cortex. None of the suggestions present convincing evidence in support. As Mollon says [22], the unique hues “remain one of the central mysteries of color science.” Despite functional imaging studies of the human brain [23], [24], neurophysiological studies of the monkey brain [25], [26], and recent progress in understanding the higher mechanisms of color vision [27], [28], there is no evidence of any neurones with similar chromatic tuning to unique hues. Recent literature appears not to favor the cones as a likely neural basis of unique hues, though they were so considered in early color vision models, which persist today in the form of the so-called Standard Model (e.g., as used in most colorimetry-based color appearance models [9]): opponent-color chromatic response functions are modeled/calculated directly from combinations of cones, though without direct physiological evidence. In sum, the retina, LGN, and cortex have all been variously proposed as the neural basis of unique hues, with no wide agreement.

This paper demonstrates that an extremely common type of subcortical neurone, the cone photoreceptors numbering some 5 million over the retina, are exactly tuned to the spectral (*b*, *g*, and *y*) opponent-color chromatic response functions, which carry three unique hues. The fourth opponent-color chromatic response *r*, which carries the red unique hue, is largely nonspectral (outside the spectrum) and thus does not correspond to a single cone; it is presumably produced by a combination of *S* and *L* cones.

To demonstrate the physiological basis of unique hues it is first required to confirm that their psychophysical or behavioral basis is the opponent-color chromatic response functions, so these may then be compared to potential physiological bases.

### Hypothesis

With a view to to clarifying the physiological basis of unique hues, the aim of this paper is to test the following hypothesis: That cone receptor spectral sensitivity functions (or cone response functions) and opponent-color chromatic response functions are closely correlated. The tests will employ previously published well-known data sets, experimental and psychophysical. Cone responses and chromatic responses will both be compared in equal formats: in linear form, as positive curves normalised at 1.0 maximum response. (“Correlate” is used in this paper in both the qualitative and - where Pearson correlation coefficients are calculated – the quantitative sense.)

Tests will include the following comparisons: (1) comparison of wavelength peaks of cone response curves with three (*b*, *g*, *y*) of the four unique hue response curves; the latter will be treated in two data groups, as vision models and hue cancellation experiments, with emphasis on the latter empirical data; (2) comparison of the wavelengths of crossovers (or intersections) between cone response curves and between chromatic response curves; and (3) comparison of curve shapes (or math functions) of cone responses with those of *b*, *g*, *y* chromatic responses.

Tests will include the following predictions: (1) prediction of *b*, *g*, *y* chromatic response wavelength peaks from cone sensitivity data; and (2) prediction of both cone response curves and *b*, *g*, *y* chromatic response curves by the same curve fitting equations.

The results indicate that close correlation exists, confirming the hypothesis. Correlation alone does not prove a causal relationship, but in this case causality is demonstrated by other factors. In confirming the hypothesis, valuable information has been gathered on arguably the most important physiological component (cones) and arguably the most important psychophysical component (unique hues) of color vision.

## Psychophysical (Behavioral) Basis of Unique Hues

Opponent-color chromatic response functions are the most likely candidate for the psychophysical basis of unique hues. The literature indicates that each unique hue is stimulated by its respective chromatic response function, but the unique hue can only be perceived at the one wavelength where the other opponent color mechanism (say, the *y-b*) is in equilibrium, that is, is at null response (Figs. 1 or 2). This logically allows two alternatives: (1) that each chromatic response curve stimulates one hue, its own unique hue, over all its wavelength range, or (2) that each chromatic response curve represents a range of hues including the unique hue. Alternative (1) represents the hypothesis to be tested in this section. If confirmed, each chromatic response curve, representing its respective unique hue over all its wavelength range, would clearly represent the psychophysical basis of that unique hue.

The hue cancellation literature [6], [11], [29], [30] nowhere states a chromatic response function represents one unique hue rather than a range of hues, but nor does it explicitly deny the possibility. The experimenters generally speak of a common “hue component” (e.g., blue), but not a unique hue, throughout the wavelength range of a chromatic response. Their habit is to refer to response peaks, e.g., as peaks of “blueness” or “redness”, inferring but not stating unique blueness or redness. The experimenters frequently refer to any one function as containing, for example, a “blue component.”

Before describing two methods of determining the hue(s) of each chromatic response function, two points should be noted. First, note from Figs. 1 or 2 that the wavelength peak of each chromatic response curve is some distance from the unique hue wavelength. Given that the peak of any response function is its most important single feature, the substantial difference between the unique hue wavelength and the curve peak in both vertical and horizontal dimensions indicates the unique hue location does not represent the prime purpose of the chromatic response function. This implies the unique hue wavelength is of secondary significance to the chromatic response function, and that the response function has evolved for some other purpose than to promote the unique hue specific wavelength. If the chromatic response consists of just one hue (a unique hue) over all its wavelength range, the curve peak represents the strongest effect of that hue in color mixture and color appearance. But if the chromatic response consists of various hues, then the response peak may represent some other hue than the unique hue. Such would present a most unexpected complication to color science.

Second, note that in color mixture there is no need for a chromatic response curve to vary its hue in order to achieve color mixture. As shown in hue naming and hue prediction experiments [6], [10], [11], [31], mixing the *g* response curve, for example, with the overlapping *b* response, produces all possible variations of blue-green hues from bluish-green to greenish-blue. Similarly, overlapping chromatic response curves mix the full variety of hues over the spectrum (Fig. 1) or the full hue cycle (Fig. 2), without any need for hue variation within each chromatic response function.

**Figure 3:**
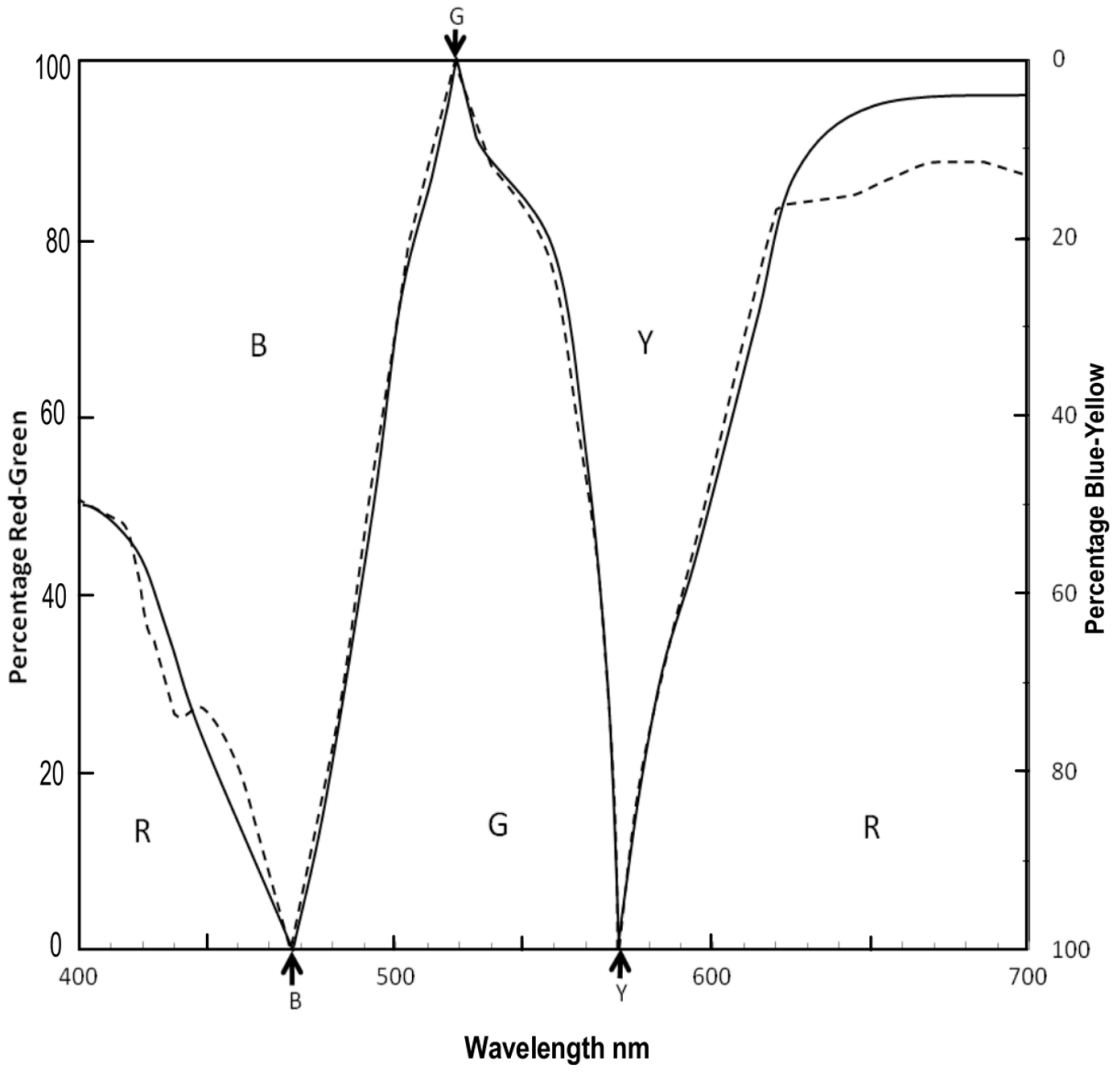
Hue naming and hue prediction data. Re-drawn from Werner & Wooten 1979. Black solid lines: Hue naming data for three subjects (mean). Black dotted lines: Hue prediction for same three subjects using Hurvich & Jameson vision model [7]. Arrows indicate unique hue loci at 100% R or G (left ordinate), and B or Y (right ordinate).

The question of “Whether each chromatic response curve represents only one hue?” can be deduced by (at least) two methods from previous hue prediction models and hue naming experiments. The first method concerns the formal meaning of the algebraic terms *b*, *g*, *y*, *r*, as symbols in equations. Werner & Wooten [11] compared their hue naming experiment for three subjects with Hurvich & Jameson's hue prediction model [6], applied by Werner & Wooten to the same three subjects. As Fig. 3 shows, the results are in close agreement, indicating the accuracy of the hue prediction model. The results agree with other hue prediction and hue naming data.

Hurvich & Jameson [6] predicted spectral hues by means of hue coefficients derived from chromatic response functions. Their model has been stated [11] as:
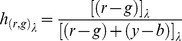
(1a)

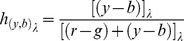
(1b)The symbol *h*_(*r*, *g*) λ_ means “the hue coefficient for *r* or *g* of a given wavelength”. That wavelength applies also to all the hues *r*, *g*, *y* or *b* in the equation. The hue coefficient, or proportion of a particular *rygb* hue relative to all four hues sampled at that wavelength, is extracted from the equation, for say *b*, as *b/(b*+*g+y+r)*. Hurvich & Jameson's hue coefficient method operates very successfully (Fig. 3) and its accuracy is indicated by its close agreement with hue naming experiments (Fig. 3).

But what does *b*, *y*, *g* or *r* represent? In formulaic terms the symbols *b*, *y*, *g*, and *r* have precise mathematical meanings: each is a specific quality that remains constant in the equation independent of wavelength. Only the hue coefficient (a quantity) is variable by wavelength. In terms of color, therefore, each of *b*, *y*, *g* or *r* represents a single hue that is constant over the wavelength range of the respective chromatic response function. That hue can only be determined by its color appearance at the one wavelength where it is perceivable without being overlapped and admixed with another chromatic response curve, that is, when the other opponent-color system is in equilibrium. This occurs only for the unique hues; therefore each chromatic response over its wavelength range represents a unique hue.

The above applies also to any other equations that depend on a constant hue quality in each chromatic response function *b*, *g*, *y* or *r*. For example, returning to Hurvich & Jameson's seminal work, consider the transformation equations from cones (Short-wave, Medium-wave, Long-wave, or *SML*) to the chromatic responses (the equations are here abbreviated by omitting coefficients):
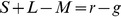
(2a)

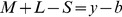
(2b)Similarly, transformation equations to convert CIE [32] tristimulus values XYZ to the opponent-color chromatic responses also employ the qualities *b*, *g*, *y*, and *r*.

All such equations would be mathematically invalid unless the qualities *b*, *g*, *y*, and *r* were each constant throughout the wavelength range of the corresponding chromatic response function. The same applies to *SML* cone responses and XYZ tristimuli: while response amplitudes can vary across any one response, the quality of the response cannot vary. In the case of *SML* cone responses, they obey the principle of univariance [33] in which varying the wavelength of light incident upon one cone varies the cone response amplitude but does not vary its wavelength or the resultant hue.

A second method of defining the hue(s) of each chromatic response function is by analyzing the technique of hue naming. Werner & Wooten's hue naming data (for monochromatic stimuli) closely agree with hue prediction data, shown in Fig. 3. The observers were instructed to describe the percentage of red, yellow, green, or blue, in a given test color. For any test color, only two hue names were allowed. Now, the instructions to observers would be unworkable unless it were implicitly assumed (by experimenters and observers) that the red, yellow, green, or blue hues being described were each a constant hue at any wavelength over the spectrum. This constant hue can only be observed at one wavelength, that of the 100% hue determined by the color naming experiment.

Agreement between the two methods strengthens the validity of their results. All methods depend on the constant meaning of a term, whether verbal in experiments or algebraic in formulas..

### Results Summary

It is concluded that each chromatic response curve represents only one hue over its wavelength range: a unique hue. In other words, each chromatic response may respond to varying wavelength stimulus by varying the amount of chromatic response but not the hue. This result has a similar logic to that of the (physical) principle of univariance mentioned above. Applied to the chromatic response functions, they may be said to obey a psychophysical version of the principle of univariance.

Hence each *b*, *g*, *y*, or *r* response curve defines, and may properly be termed, a *unique hue chromatic response function*. The result clarifies the role and importance of the chromatic response functions, and confirms the opponent chromatic response functions are the psychophysical basis of unique hues. There is really no other reasonable candidate. These functions can now be compared with physiological functions that may potentially represent the physiological basis of unique hues.

## Physiological Basis of Unique Hues

Previous analyses have generally employed more complex methods [17], [18], [34], but here, data are presented and quantitatively compared in simple terms of function wavelength peaks and function curves graphed to linear scale and normalized for ease of comparison. Customarily cone response curves are shown in log form with normalised curves, often to an *x*-axis of wavenumber (frequency). In contrast, chromatic responses are customarily shown in linear form, with curves arranged as opposed negative and positive curves (the conventional signs are arbitrary) and not normalised, to an *x*-axis of wavelength. Such practices made cone responses and chromatic responses difficult to compare and tended to obscure the similarities.

### Color Vision Models

**Figure 4:**
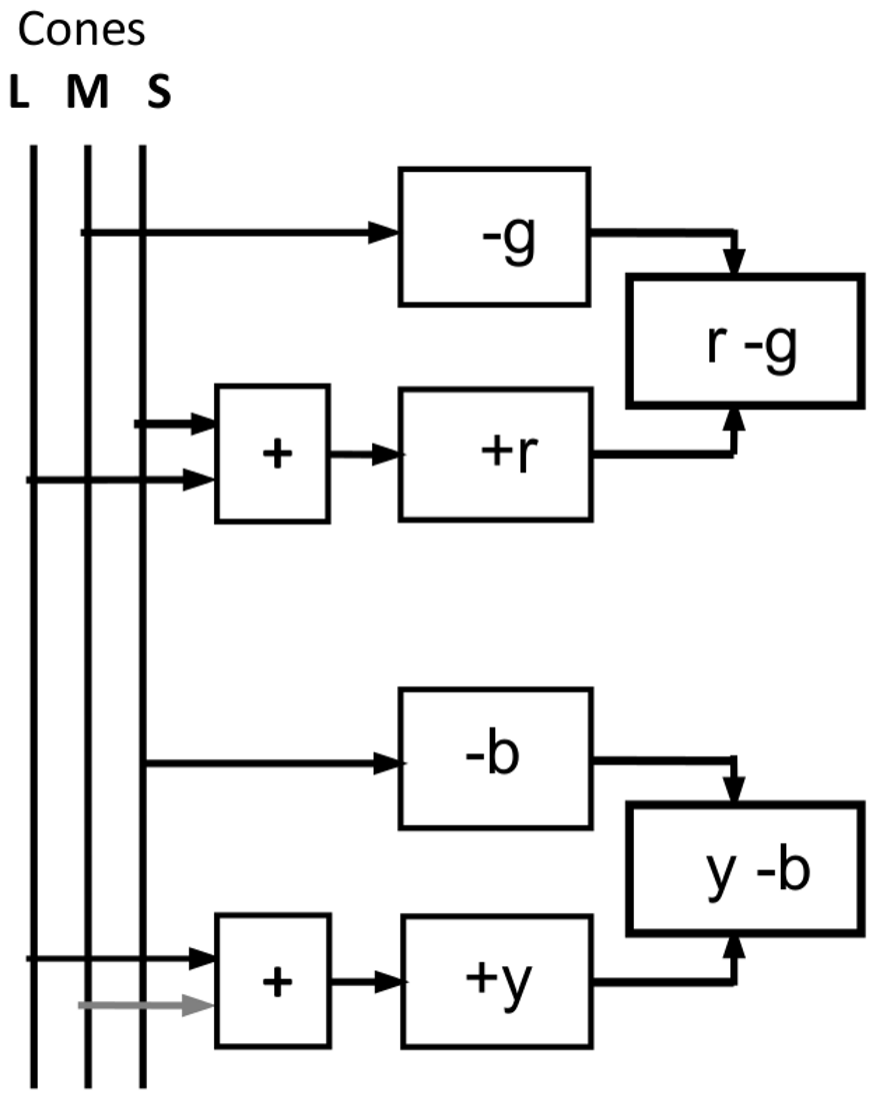
Standard model of color vision and opponent-color process. This schema shows the visual process from LMS cone sensitivities to unique hue chromatic response functions *r-g*, *y-b*. Removal of grey line (*M* input to *y*) converts the schema to to the hypothetically simplest effective model where *S = b*, *M = g*, *L = y*; response curve *r* requires inputs from both *S* and *L*, i.e., *S+L* = *r*.

Color vision models are surveyed to find the simplest and/or most effective transformation between stages. Simplicity in visual process translates to neural economy, favoured by evolution [35]. The early multistage color vision models (5,6) conceived the opponent-color chromatic response functions, *r-g* and *y-b*. These functions were further developed by later models [8], [9], [11], [36], [37], [38], [39], [40], [41] and were broadly confirmed by hue cancellation experiments (below). The models derive chromatic responses from combinations of cone responses as shown in Fig. 4, a schematic diagram of the generally accepted Standard Model described and employed by Fairchild [40] in color appearance models, modeling the visual process from *SML* cone sensitivities to chromatic response functions *r-g*, *y-b*.

Table 1, below, shows wavelength data on the above vision models. Note that most agree on the approximate wavelength peaks, whose means are 442, 530, 567, with two spectral peaks for the red function about 440 and 615 nm. These peaks form the *r* function which extends from one end of the spectrum to the other over the nonspectral hues (reds and purples, the latter formed by overlapping *r* and *b* curves; see Fig. 2). Interestingly the two *r* peaks are confirmed by previous colorimetric calculation of optimal compound color stimuli [13], [14]: the wavelengths 442+613 nm±1 (representing blue and red components of nonspectral colors) are always the optimal pair of components in additive mixing of all nonspectral colors in all standard illuminants.

**Table 1:** Opponent-color chromatic response peak wavelengths of various color vision models.

Chromatic response peaks, nm				
*b*	*g*	*y*	*r*	References
445	530	555	440, 610	Judd [5]
445	530	560	440, 610	Hurvich [6]
440	530	560	nil, 610	Ingling [36] (version 1)
440	530	560	440, 610	Ingling [36] (version 2)
455	530	580	440, 610	Werner [11]
445	530	565	445, 615	Hurvich [9] (neutral)
435	530	565	nil, 610	Guth [37]
435	530	570	440, 620	Guth [39]
440	530	599	440, 610	De Valois [38] (stage 3)
440	530	560	440, 610	Fairchild [40] (“standard”)
442	535	568	442, 613	Pridmore [41]
442	530	567	441, 612	*Means*

These data were psychophysically estimated but usually guided by experimental hue cancellation data (Table 2). Only the first author of indicated references is listed. The *r* curve has two spectral lobes (see Fig. 1) in short and long wavelengths, always about 442 and 613 nm from hue cancellation data.

It is worth noting the two spectral *r* peaks (probably derived from *S* and *L* cones as in convention, Fig. 4) are found in hue cancellation experiments to represent the same red hue although they arise from two different cones. Presumably the physiology allocates both the *S* and *L* cone inputs to creating one chromatic response function *r*, and therefore to one chromatic sensation, unique red.

The Standard Model of color vision combines inputs from all three cones to each of its opponent-color functions, *r-g* and *y-b*, as schematized in Fig. 4. A different combination used in a recent model [41] inputs *S* cone directly to *b* chromatic response, *M* directly to *g*, and *L* directly to *y*, from the author noting the similarity of wavelength peaks in the data. The Standard Model similarly inputs *S* cone directly to *b* chromatic response, and *M* directly to *g*, but inputs *L*+*M* to *y* chromatic response. This latter combination seems based on early (but long-held) beliefs in the *RGB* (rather than *LMS*) cones and therefore that *R*+*G* = yellow. The combination is somewhat surprising because *L*+*M* peak wavelengths (say 565 and 530 nm) give a mean 547 nm, well short of the commonly agreed wavelength peak about 565 nm for *y* chromatic response. Hence, one might reasonably expect that the cone input to *y* chromatic response should be from *L* alone. This would change the Standard Model to direct correspondence such that *S*≈b, *M*≈g, and *L*≈y. Such a relationship, though little different from the Standard Model, would clarify the simplicity of the relationship between cones and unique hue chromatic responses. This possibility is explored below. Note that some models in Table 1 give only a schema of transformation (like Fig. 4) while others give detailed transformation equations, e.g., [6], [7], [8], [9], [11].

Table 1 lists two models which lack a short-wavelength lobe to the *r* chromatic response, which are now widely accepted to be flawed since they are unable to predict reddish-blue hue (violet) in the short-wavelength end of the spectrum. Guth later admitted this shortcoming of his 1980 model [38] and corrected it in his 2002 model [39]. Ingling [36] claimed his model was justified by conflict between hue cancellation data and direct hue matching [42] but his model was later [43] shown to be erroneous by CIE colorimetry; in effect, Ingling was using a very desaturated, almost flat-lined, version of the *b* stimulus curve. Omitting these flawed models, the simplest effective models in Table 1 seem to be those in Refs [8], [9], [41]. These models minimize or omit *M* cone input to *y* chromatic response (see Fig. 4 caption). Though Hurvich and Jameson did not purposely equate their *bgy* chromatic response curves to *SML* cone response curves, the two sets closely agree as will be shown later below.

### Cone Responses: The Experimental and Psychophysical Data

While the above models were developing, experimental research of cones and opponent chromatic responses proceeded. Microspectrophotometry showed absorption spectra for the three cone pigments overlap widely but peak in three different parts of the spectrum at about 420, 530, and 560 nm [44], [45], [46]. Earlier data in the 1960s, e.g. [47], were probably less accurate due to methodology. These absorption spectra shift to longer wavelengths (about 445, 535, 565 nm) in cone spectral sensitivities *in vivo* due to transmission properties of the ocular media, e.g. yellowish lens and macular pigment.

Data sets on cone spectral sensitivities [48], [49], [50], [51], [52], [53], [54], [55], [56], [57] are mostly in broad agreement and are shown in Table 2. The table includes all or most of the available data. Most are psychophysically estimated from experimentally determined RGB color matching functions [32] and experimental data on cone absorptions. The Stiles data [57] represent the difficult method of field sensitivities, included to show a different method from the usual. Table 2 shows spectral sensitivity peaks of *SML* cones are grouped in three distinct areas of the spectrum, about 440–450, 530–540, and 560–570 nm.

**Table 2:** Wavelength peaks of human cone spectral sensitivities and of opponent chromatic responses from hue cancellation experiments.

Cone sensitivity peaks from psychophysical and experimental data				Opponent-color chromatic response peaks from hue cancellation experiments				
*S*	*M*	*L*	References	*b*	*g*	*y*	*r*	References
440	540	565	Smith [48]	435	530	550	440, 620	Jameson [6]
445	540	560	Judd [52]	440	530	555	440, 610	Romeskie [29]
444	527	571	Estevez [49]	455	525	582	443, 610	Werner [11]
450	540	560	Wyszecki [53]	447	535	565	440, 610	Takahashi [30] #
440	540	565	Wyszecki [54]	445	530	565	445, 615	Takahashi [30] †
444	530	571	Wyszecki [55]	445	530	564	440, 610	Takahashi [30] *
438	533	564	Dowling [56]	445	535	570	442, 610	Takahashi [30] #L
445	535	570	Vos [57]	445	535	565	442, 610	Takahashi [30] †L
440	530	560	Stockman [50]	440	540	560	460, 610	Fuld [58]
440	540	565	Stockman [51]	440	525	560	440, 610	Kulp [59]
*443*	*535*	*565*	*Means*	*444*	*532*	*564*	*443, 612*	*Means*

These data represent, or are calculated from, experimental data. Under References only first authors are listed. Wyszecki (55), (56), and (57), refer respectively to Wyszecki & Stiles (1967) Konig-type fundamentals, Vos & Walraven (1978) fundamentals, and Stiles (1953, 1959) field sensitivities for π_1,_ π_4,_ and π_5_ mechanisms. In Fuld (1991), and in Kulp & Fuld (1995), data are given at large (20 nm) intervals so mean data for the 3 or 4 subjects respectively were plotted, curves drawn and peaks interpolated to nearest 5 nm as listed above. Takahashi (30) gives several troland levels for 8700 K color temperature and 2 subjects:

#denotes 50 td,

†is 500 td, and

*is 5000 td.

Two troland levels are shown for 5200 K and 1 subject:

#Ldenotes 50 td,

†Lis 500 td.

### Chromatic Responses: The Experimental Data from Hue Cancellation

Color vision models generally estimate their opponent chromatic responses *y-b*, *r-g*, from CIE 1931 *XYZ* tristimulus values [32] by linear and simple nonlinear transformations. The modelers of these functions (Table 1) presumably first checked them with the available hue cancellation data. Table 2 lists the few available data sets on hue cancellation experiments [6], [11], [29], [30], [58], [59] and particularizes the detailed study by Takahashi et alia for various luminance levels and two color temperatures of illuminant. (Note the tabled Jameson data refer to one subject J, omitting subject H as suspect with overly similar *g*, *y* wavelength peaks). These data broadly agree with most vision models' response peaks in Table 1, indicating that most modelers were guided by the experimental data. Tables 1 and 2 closely agree on the two spectral peaks of the *r* function, about 440–445 and 610–615 nm. As already mentioned, these two *r* lobes probably derive from *S* and *L* cone inputs as in the Standard Model.

The *bgy* peaks in Table 2 are grouped in three distinct areas of the spectrum, about 440–450, 530–540, and 560–570 nm. These groupings are the same as the *SML* cone sensitivity data also in Table 2. Further, the table's means for the *SML* cones (443, 535, 565 nm) and for the *bgy* chromatic responses (444, 532, 564) are in notably close agreement. Both sets are 443.5, 533.5, 564.5±1.5 nm. The Pearson correlation coefficient (*r*) between the two sets of means is extremely high at 0.9998 (see Table 3), indicating the two sets are identical for practical purposes. (Reduced to three decimal places, 0.9998 becomes 1.0 perfect correlation).

**Table 3:** Pearson correlation coefficients (*r*) between sets of curves or sets of wavelength peaks.

Correlation coefficients			
*Correlation between cone response curves and chromatic response curves:*			
*S* and *b*: 0.989	*M* and *g*: 0.870	*L* and *y*: 0.986	Mean 0.948
*Between predicted curves (* *Eqs. 4* *–* *6* *) and cone response curves:*			
Eq. 4 and *S*: 0.981	Eq. 5 and *M*: 0.985	Eq. 6 and *L*: 0.996	Mean 0.987
*Between predicted curves (* *Eqs. 4* *–* *6* *) and chromatic response curves:*			
Eq. 4 and *b*: 0.985	Eq. 5 and *g*: 0.931	Eq. 6 and *y*: 0.986	Mean 0.967
*Between SML and bgy sets of wavelength peaks (* *Table 2* *):*			
0.9998			
*Between SML and bgy sets of wavelength peaks (* *Tables 1* * & * *2* *):*			
0.9992			
*Between SML and bgy sets of wavelength peaks (* *Table 1* *):*			
0.9985			
*Between SML and bgy sets of curve crossovers (* *Table 4* *):*			
0.9965			

Items (1) to (3), below, correlate sets of curves where data were sampled at 5 nm intervals in the range 400–640 nm. For items (4) to (7), all peak wavelengths, not merely the means, were taken to calculate *r*. Coefficients are listed for: (1) correlation between cone sensitivity curves [51] and opponent chromatic response curves [9] as arranged in Fig. 6; (2) correlation between predicted curves from Eqs. (4)–(6) and *SML* cone curves in Fig. 7; (3) correlation between predicted curves from Eq. (4)–(6) and *bgy* chromatic response curves in Fig. 7; (4) correlation between *SML* and *bgy* sets of wavelength peaks (*bgy* from hue cancellation data), Table 2; (5) correlation between *SML* and *bgy* sets of wavelength peaks from all data (both Tables 1 & 2); (6) correlation between *SML* (Table 2) and *bgy* sets of wavelength peaks (*bgy* from vision models), Table 1; and (7) correlation between *SML* and *bgy* sets of curve crossover wavelengths from Table 4, columns 3 and 4.

The correlation coefficient for the same *SML* peaks and the mean of all *bgy* wavelength peaks (442.9, 530.9, 565.65 nm) in the available data (say, Tables 1 and 2) is very similar at 0.9992. Correlation of the same *SML* peaks with the *bgy* mean wavelength peaks for color vision models only (Table 1) is rather lower at 0.9985 (Table 3), as may be expected.

The agreement demonstrates an extremely close relationship between *S* and *b*, *M* and *g*, and *L* and *y*. One-to-one correspondence gives the two sets the same cardinal number, indicating the highest correspondence [60]. It surprises that this obviously close association of *SML* responses to *bgy* chromatic responses, even without correlation coefficients, has gone so long unpublished (despite my repeated attempts with eight journals over 22 years since 1991) since the wavelength peaks are independent of the linear or log form of the curves.

### Graphical Comparisons

**Figure 5:**
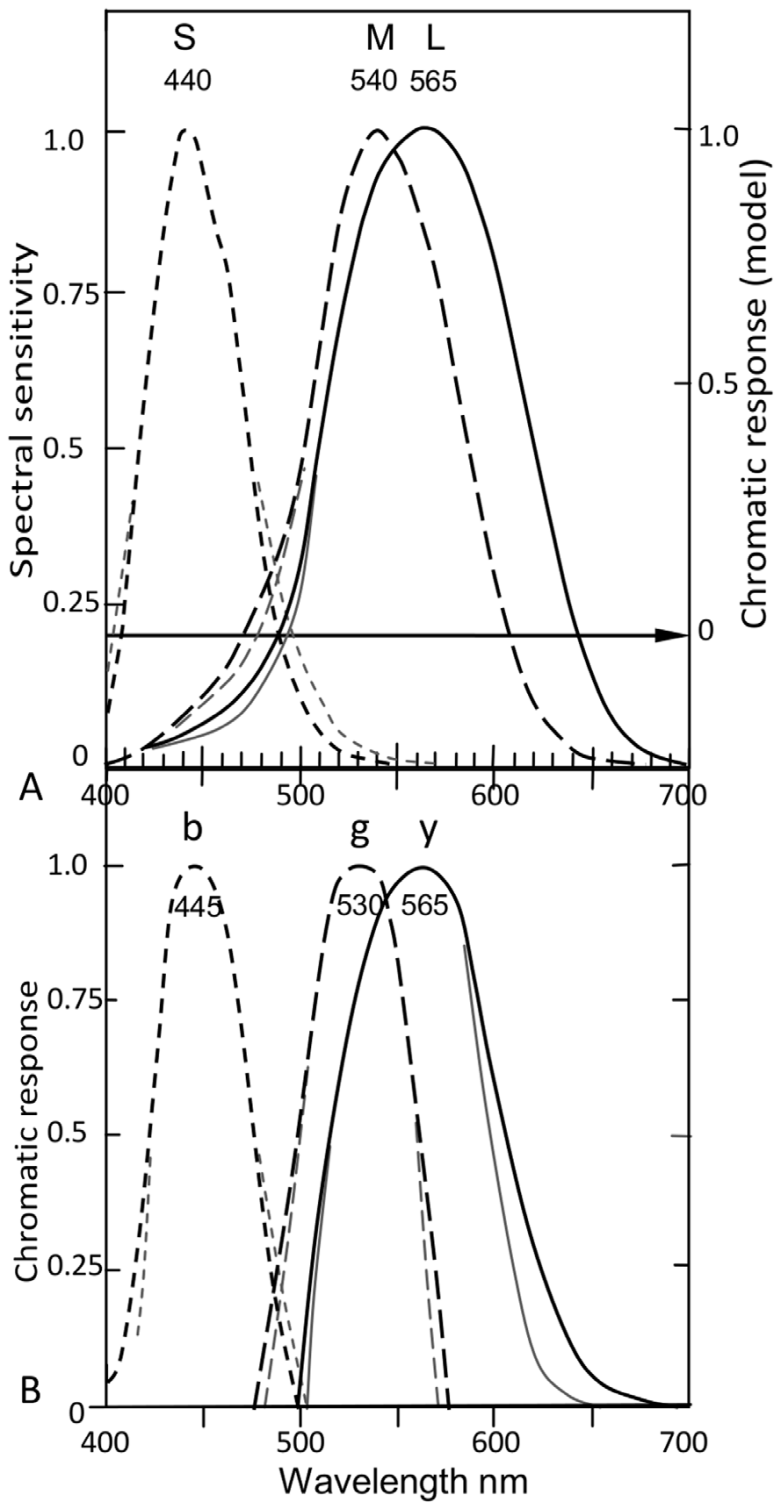
Cone and chromatic response curves normalized at 1.0 response. **A**. Black lines: Cone sensitivity estimates of Stockman & Sharpe [51] with log values converted to linear. Gray lines: Cone sensitivity estimates of Smith & Pokorny [48] where they differ much from the former. Arrowed horizontal line from *S* and *L* curves' intersection (predicting *b* and *y* curves' intersection) indicates null response of new chromatic response model at right *y*-axis. **B**. Black lines: Opponent chromatic response curves from Hurvich [9] in Fig. 1. Gray lines: Chromatic response curves from hue cancelation experiments of Takahashi [30] where they differ much from the former, for 8700 K and mean data for 500 and 1580 td (say 1000 td), mean of two subjects.

The agreement of tabulated data for cone responses and *bgy* chromatic responses is supported graphically in Fig. 5, which compares two well-known sets of cone data with two well-known sets of chromatic response data. Fig. 5A shows cone sensitivity data from Stockman and Sharpe [51] in black and from Smith and Pokorny [48] in gray, where the latter curves differ significantly from the former (only at lower values); the wavelength peaks are identical. Fig. 5B shows chromatic response curves for *bgy* (all positive and normalized at 1.0 response) in black from Hurvich's 1981 model [9] in Fig. 1 (which was closely guided by hue cancellation data) and from the Takahashi et al. hue cancellation data [30] in gray, only where the latter curves differ significantly from the former; the wavelength peaks are identical. Wavelength peaks in Fig. 5A are similar to those in Fig. 5B, and may be given as 442.5, 535, 565±5 nm. The weakest similarity is between *M* and *g* peaks at 540 and 530 nm due to the particular data sets chosen, but the mean data are more relevant at 535 and 532 nm (Table 2). The agreement is not only in wavelength but in curve crossovers/intersections and particularly curve shapes: *S* and *b* are the narrowest curves (at say 0.5 chromatic response, y-axis), *M* and *g* the next narrowest, and *L* and *y* the broadest. These different areas of agreement demonstrate one-to-one correspondences in several aspects between the two sets.

These graphical similarities, so obvious in Fig. 5, have been obscured by cone sensitivities customarily being presented in log scale and normalized, and chromatic responses in linear scale and not normalized, and further, graphed as opposed-sign curves (Fig. 1).

### Model

It is required to more carefully test the degree of equality between *SML* responses and *bgy* responses. A model suits this purpose. Eq. 3 is a theoretical model of relations between *SML* cone sensitivity curves and *bgy* chromatic response curves, where≈means ‘approximately equals.’
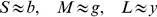
(3)


Within this model, though not directly involved, the *r* curve is defined as in the Hurvich 1981 model with wavelength peaks near 442 and 613 nm; these lobes, located at opposite ends of the spectrum, can not derive from one cone but only from two cones *S* and *L*.

The model may be tested by predicting chromatic response curves from given cone sensitivity curves, or the reverse. The given cone sensitivity curves will be those in Fig. 5A (selected as representative of such curves) shifted laterally such that peaks align with respective mean wavelength peaks in Table 2. The *bgy* curves to be compared with the prediction are the Hurvich curves in Fig. 5B (selected as representative) similarly shifted to the respective mean wavelength peaks in Table 2. In Fig. 5, the cone curves continue below the *S* and *L* curves' intersection whereas the corresponding *b* and *y* response curves terminate at their intersection representing null response level. To normalize Figs. 5A and 5B for comparative analysis, the same null response level is assumed of the intersection of *S* and *L* curves since they are the original cones [61] setting up primate vision; the later *M* cone presumably adopted the existing null response level. Accordingly, the arrowed horizontal line in Fig. 5A indicates null response level at the right *y*-axis, whose scale from 0 to 1.0 becomes the model of chromatic response within the Eq. 3 model.

**Figure 6:**
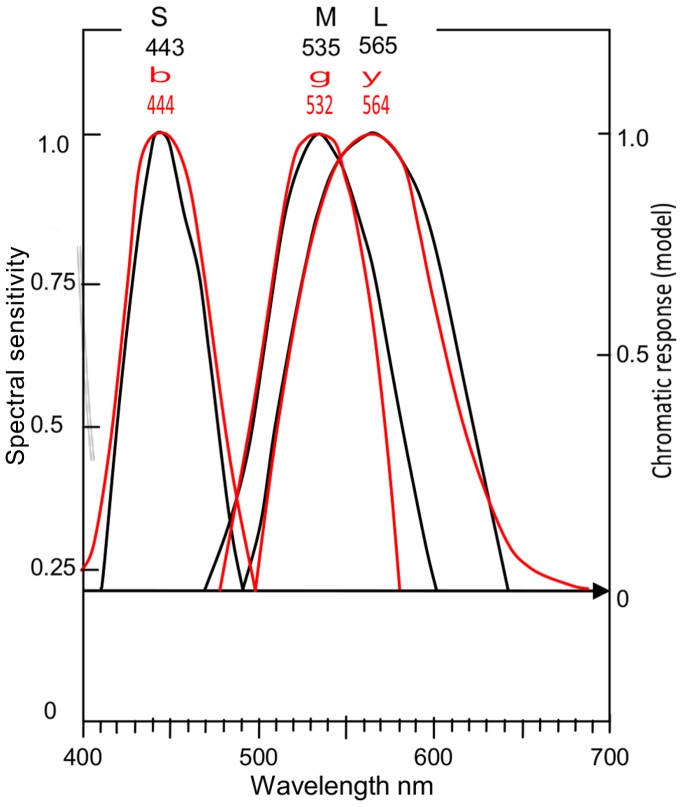
Cone sensitivities and opponent chromatic responses overlaid. As Fig. 5, but with Fig. 5B *bgy* chromatic response curves (in red) overlaid on Fig. 5A *SML* cone curves (in black) and fitted to right *y*-axis from 0 to 1.0 chromatic response. The 0 response level derives from the arrowed line marking the level where *S* and *L* curves intersect. Both sets of curves are shifted laterally to align with their mean wavelength peaks (per Table 2) as labeled. Cone curves are shown only ≥ the level, arrowed, where *S* and *L* curves intersect, forming hypothetical null response in the chromatic response model on right *y*-axis.

The response scale (*y*-axis) distances from 0 to 1 in Figs. 5A and 5B need equalizing. For this reason and for direct comparison, Fig. 6 shows Fig. 5B and its *bgy* curves overlaid on Fig. 5A, and the response scales equalized. The *SML* curves (in black) are shifted laterally to their mean wavelength peaks (Table 2). These curves (and their math functions) now represent the model's prediction of *bgy* response curves. To test the prediction, the *bgy* curves (in red) are similarly shifted to their mean wavelength peaks. Clearly, the two sets of curves are approximately equal.

The correlation coefficients (Pearson product moment) for the three pairs of curves are quantified in Table 3; their average is 0.95, a very large or high degree of positive correlation (over 0.5 is regarded as “large”). Coefficients were calculated from data at 5 nm intervals from Ref. [51] and from Ref. [9] curves, after normalising and digitising from Fig. 6. Minor differences exist but the generally close correlation of overlaid curves in Fig. 6 confirms the prediction (Eq. 3). If, in Fig. 6, the Hurvich chromatic response curves were instead laid over the Smith and Pokorny cone functions (gray lines in Fig. 5A), agreement would be slightly improved at lower response levels.

The term “cone responses,” used in this paper, is a rather loose term for cone sensitivities, since the cone electrical output responses (known as cone action spectra) may in principle differ from the cone sensitivities in curve shape. However use of the term is supported by data on cone action spectra. After allowing for absorption by optical media, the corrected action spectra match quite well with the cone sensitivity data [62].

### Curve Fitting

**Figure 7:**
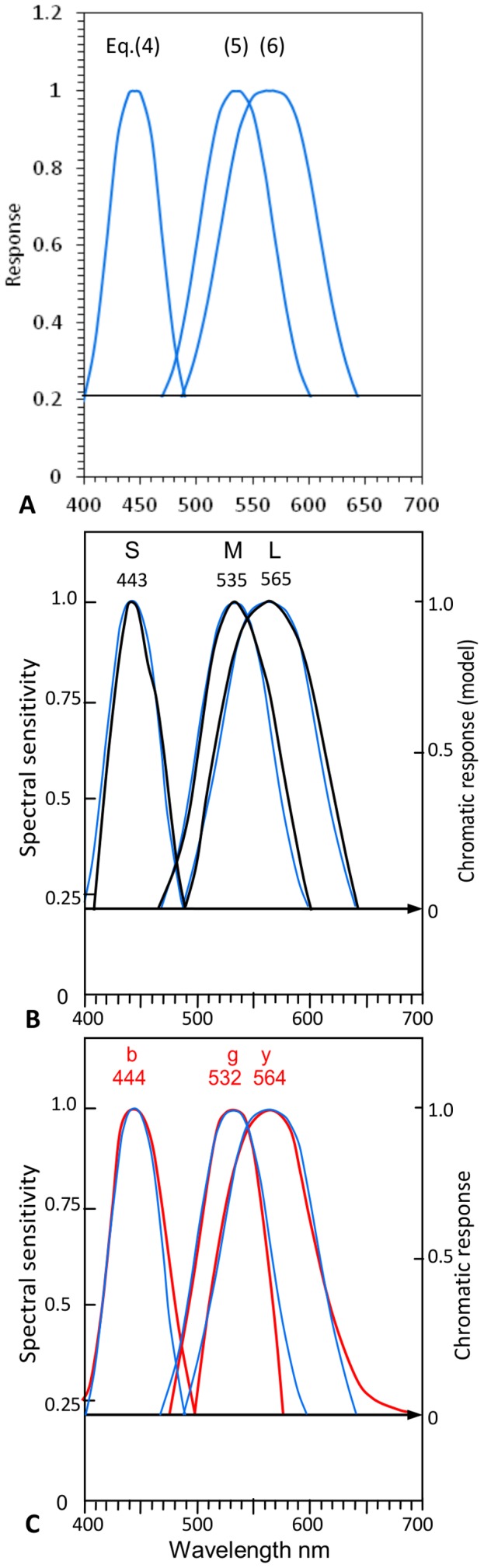
Curve fitting prediction of cone sensitivities and opponent chromatic responses. **A**. Three formulaic curves [from Eqs. (4)–(6)] predicting *SML* cone curves. Their wavelength peaks are nominally 445, 535, 565 nm but can be shifted horizontally to any wavelength without changing shape. **B**. The formulaic curves compared to *SML* cone response curves (taken from Fig. 6) by overlapping the latter wavelength peaks (as labeled). **C**. The formulaic curves compared to *bgy* chromatic response curves (from Fig. 6) by overlapping the latter wavelength peaks (as labeled in red). The equations predict cone response curves and chromatic response curves about equally well, at correlation coefficients of 0.99 and 0.97 respectively (Table 3).

To quantify agreement in *curve shape* between cone responses and chromatic responses in Fig. 6, a curve fitting exercise determined Eqs. (4)–(6) which plot the curves shown in Fig. 7A. These have closely similar shapes to the curves *S* and *b*, *M* and *g*, *L* and *y*, as shown in Figs. 7B and 7C.
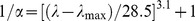
(4)

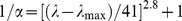
(5)

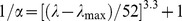
(6)where λ represents wavelength (or *x*) on the *x*-axis of Fig. 7, and λ_max_ represents the curve's wavelength peak; and α represents Response amplitude (spectral sensitivity on left *y*-axis).

Fig. 7B shows cone response curves (in black) taken directly from Fig. 6. Fig. 7C similarly shows the unique hue chromatic responses (in red) taken from Fig. 6. The latter curves are fitted vertically to the right *y*-axis between 1.0 max response and null response (arrowed horizontal line, as in Fig. 6). Alignment of curves with their respective mean wavelength peaks (just as in Fig. 6) allows a better perspective of average placement of curves and their interrelationships, e.g., curve crossovers. Quantitative data for crossover wavelengths are listed in Table 4, discussed later below.

**Table 4:** Interrelationships between curves.

Crossovers	Cones Fig. 5	Cones Fig. 6	Chromatic responses
Crossover λ of *S* & *M*:	484 (488)	485	
Crossover λ of *b & g:*			486
Crossover λ of *S* & *L*:	490 (495)	492	
Crossover λ of *b & y:*			498
Crossover λ of *M* & *L*:	550 (550)	547	
Crossover λ of *g & y:*			547

Crossover or intersection wavelengths (λ) nm of *bgy* opponent chromatic response curves (last column) as shown in Fig. 6, and crossover wavelengths of *SML* cone curves from: (a) Fig. 6, where *SML* peaks are shifted laterally to mean wavelength peaks in Table 2; and (b) Fig. 5 from Stockman & Sharpe (51), and from Smith & Pokorny (48) in parentheses.

The formulaic curves in Fig. 7A are re-plotted in Fig. 7B such that the wavelength peaks [λ_max_ entered in Eqs. (4)–(6)] align with the cone response curve peaks at their mean wavelengths (labeled in black). The same formulaic curves are similarly shown in Fig. 7C so they align with the chromatic response curve peaks also positioned at their respective mean wavelengths (labeled in red).

This form of equation employs a function (λ-λ_max_) similar to Dartnall's nomogram or log formula [63] for standard shape of cone absorption spectra, but plots a symmetrical curve in linear form, unlike Dartnall's asymmetrical curve in log. Eqs. (4)–(6) have a common essential form, but need the variations of Eqs. (4)–(6) as a function of wavelength to match the target curves. Dartnall's standard formula was similarly found to require modifications to closely fit the target data [64]. All three formulaic curves are of the same algebraic type, and will predict any curve whose λ-λ_max_ are entered into the equation. Each equation contains two free parameters, determined by iterative trial and error to best fit both sets of curves, and predicts any number of data points, as shown in Fig. 7.

Fig. 7 illustrates the close correlation between curve shapes of cone responses and *bgy* responses. Correlation is quantified by correlation coefficients in Table 3. Both curve sets are well predicted by the same equations, though slightly more accurate for *SML* curves (coefficient 0.99). That the same equation, e.g. Eq. (4), predicts both a cone curve and a chromatic response curve with approximately equal accuracy indicates both curves have similar, possibly identical, math functions in the physiology. Figs. 7B and 7C show correlation between the predicted curve and the target curves (cone responses and chromatic responses) is notably close, averaging 0.98 correlation coefficient. Table 3 also gives correlation coefficients between cone sensitivity curves *SML* and opponent (or unique hue) chromatic responses *bgy*, respectively; their average correlation coefficient is 0.95.

### Other Correlations/Associations

The Introduction stated a third test of correlation between the two sets of curves would be quantitative comparison of curve crossover wavelengths. These wavelengths are listed in Table 4 for curve crossovers or intersections for cone curve pairs *S* and *M*, *S* and *L*, *M* and *L*; and for the corresponding chromatic response curves *b* and *g*, *b* and *y*, *g* and *y*. The data apply primarily to the two sets of curves in Fig. 6, whose curves have been shifted laterally so their wavelength peaks align with the mean wavelength peaks in Table 2. Hence these data represent average crossover wavelengths for the three crossovers in each curve set, on the reasonable assumption that the particular curve shapes of the Stockman & Sharpe *SML* cone sensitivities and of the Hurvich & Jameson *bgy* chromatic responses are approximately representative of such functions. Wavelengths for the three crossovers for each curve set are in close association at 485, 495, 547 nm±3 nm. The correlation coefficient between the two sets of crossover wavelengths (Table 4, columns 3 and 4) calculates as 0.997 (Table 3). Also shown are crossover wavelengths for the Stockman & Sharpe cone data [51] from Fig. 5 before the curves were shifted laterally in Figs. 6 and 7. As may be expected, the unmodified crossover wavelengths are less similar to the chromatic response curve crossovers.

A fourth form of correlation is worth noting. As mentioned above, each *r*, *y*, *g*, or *b* chromatic response function responds to varying stimulus intensity and wavelength by varying amplitude response but not hue, just as do cone receptor sensitivities in the principle of univariance [33]. The principle may be stated thus: the response of a photoreceptor is determined solely by the number of photons absorbed and is not sensitive to the wavelengths of those photons. In consequence there is no information in the response of any one photoreceptor about the wavelength of the light absorbed. Wyzsecki notes the principle (in logic) also applies to *RGB* color matching functions (which relate by linear 3×3 matrix to the cone sensitivities): each of them can only respond to varying stimulus intensity and wavelength by varying the amplitude of response but not by varying the hue. Varying the stimulus wavelength cannot vary the hue of any one color matching function over its wavelength range. The same applies to *XYZ* tristimuli [32], derived linearly from RGB color matching functions. In all cases (*SML* cones, *RGB* functions, *XYZ* tristimuli, and unique hue chromatic responses), each function curve is invariant in hue. Hence another aspect of agreement between unique hue chromatic responses and cone responses is that both sets of responses obey the principle of univariance.

### Results Summary

In sum, the set of *SML* cone response curves and the set of *bgy* unique hue (or opponent) response curves correlate remarkably closely (Table 3) in the following aspects:

1. One-to-one correspondence between *S* and *b*, *M* and *g*, and *L* and *y* curves (in all cases).
2. In all data sets listed in Table 2, *S* and *b* curves (at the 0.5 response level of y-axis) are narrower than *M* and *g* curves, which in turn are narrower than *L* and *y* curves. In detail, the curve shapes (or math functions) of the two sets of curves shown in Fig. 6 are approximately equal, with a correlation coefficient of 0.95.
3. The math functions of both sets of curves are predicted to similar accuracy by Eqs. (4)–(6), whose correlation coefficient with *SML* curves is 0.99 and with *bgy* curves is 0.97.
4. Mean wavelength peaks for the two sets (last row, Table 2) are practically equal at 443.5, 533.5, 564.5±1.5 nm (or 443, 533, 565±2 nm to the nearest integer), with a correlation coefficient of 0.9998.
5. The same mean *SML* wavelength peaks correlate with the aggregate means of *bgy* peaks in both Tables 1 and 2 with a coefficient of 0.9992.
6. The three curve crossover wavelengths for each of the two curve sets (Table 4, columns 3 and 4) are very similar at ±3 nm, with a correlation coefficient of 0.997.

Correlation coefficients for items 4, 5, 6 above, reduced to two or three decimal places, are 1.0. These very high coefficients (0.95 to 1.0) indicate the two sets of functions are almost identical for practical purposes.

## Discussion

The close correlation of corresponding curves in Figs. 6 and 7, quantified in Table 3, confirms the predictive model in Eq. 3: that is, *S*, *M*, and *L* curves approximately equal *b*, *g*, and *y* curves, respectively. There are no significant differences except at low response levels. Correlation of the mean wavelength peaks of the two curve sets is remarkably close by two measures (Tables 2 and 3). Given that the curve peak is the most important and effective point in a function curve, the single most cogent indicator of correlation between cone responses and unique hue chromatic responses is the very close match of the two sets of wavelength peaks. Together with other closely matched aspects, the two curve sets appear to be approximately identical math functions as particularly shown by Eqs. (4)–(6). The correlation indicates a set of subcortical neurones, the *SML* cones, are precisely tuned to the spectral unique hue chromatic responses *bgy*. Cones are a specialised but common neurone numbering some 5 million across the retina. Why this tuning occurs so early in the visual process is discussed later below.

Previous searches for the neural basis of unique hues focused only on matching the unique hue wavelengths rather than the whole function curves which define unique hues. The present treatment is comprehensive and matches not only one set of 3 or 4 wavelengths with another set (e.g., Table 2) but matches whole function curves and their interrelations (Table 4).

The Introduction stated tests of the hypothesis would include the following predictions: (1) predict unique hue chromatic response wavelength peaks from cone data, and (2) predict cone response curves and chromatic response curves from the same curve-fitting equations. Prediction (1) is satisfactorily demonstrated to within ±2 nm on average, by the agreement of mean data for cone and chromatic response peaks in Table 2. Prediction (2) is satisfactorily demonstrated in Fig. 7 by the reasonably accurate prediction of both cone responses and unique hue chromatic responses by the same set of curve fitting Eqs. (4)–(6). The *r* chromatic response is mostly nonspectral and not derivable from any one cone so it presumably derives from the *S*+*L* cones as in convention (Fig. 4).

Together with comparison of data in Tables 1, 2 and 4, this completes a comprehensive testing of the hypothesis that *cone receptor spectral sensitivity functions and unique hue chromatic response functions are closely correlated*. It is concluded that the hypothesis is confirmed.

It has often been said that structure indicates function [35]. The almost identical structures and interrelationships of *SML* cone response curves and *bgy* chromatic response curves suggest an identical function: the regulation of unique hues sensation. This appears to be the functional purpose of color vision from the very start of the visual process.

Resolving the neural basis of the unique hues largely resolves an older and bigger problem also: the troubled relationship of the Young Helmholtz trichromatic theory to the Hering opponent colors theory. The relationship has long been obscured by the different numbers of categories (three cones versus four unique hues) and thus the seeming lack of any possible one-to-one correspondence between the cone types and unique hues [22]. The primary relationship and one-to-one correspondence is between the three cones and the three *spectral* unique hue chromatic responses, as shown above. This is a direct, spectral, one-to-one relationship. The secondary relationship concerns a more difficult neural construct, that of the nonspectral hues. This secondary relationship is between two cones (S and L) and two spectral response curves (peaks ∼440 and ∼615 nm, Figs. 1 and 2) which combine to form the largely nonspectral *r* chromatic response. Unique red is itself a nonspectral hue [1], [6]. The nonspectral hues of the hue cycle arise from the overlapping *r* and *b* chromatic response curves (see Figs. 2 and 3), varying from red at one spectrum end, through purples to violet at the other spectrum end. Given the opponent or complementary nature of color vision, the red and nonspectral hues arose probably [41] to oppose or complement the green hues generated by the evolving M cone as it joined the earlier S and L cones [61] to produce trichromatic vision.

### Causal Relationship

Correlation alone does not imply a causal relationship. To imply such a relationship in logic or scientific method requires the use of inductive reasoning from empirical measurable evidence to support or disprove hypotheses about causal relations [65], [66]. First, I state the hypothesis: *Cone sensitivity functions are a necessary physiological cause of unique hue chromatic responses*.

The hypothesis is designed to require (in logic terms) a deterministic rather than probabilistic or statistical causation (63), and to require a necessary cause rather than merely a sufficient cause; that is, if **X** causes **Y**, then the presence of **Y** always implies the presence of **X**, though the presence of **X** does not necessarily imply **Y** will occur. My argument or test follows.

Now, it is well known the cones photoreceptors initiate the visual process, demonstrated by numerous experiments and accepted by numerous experts [31], [40], [67], [68]. This is not in doubt. Hence it is self evident that the cones (together with other factors such as suitable color stimulus, eye, and brain) are a necessary cause of the entire visual process, including the unique hue chromatic responses. This represents deterministic causation: whenever the unique hues are perceived, they always must follow the cone responses. The reverse is not possible.

Hence the hypothesis is confirmed. It is concluded that: (1) the cones are closely correlated with unique hues, and (2) the cones are a necessary physiological cause of the unique hues. There are many necessary causes but only one known (demonstrated above) to closely correlate with unique hues and thus be a possible physiological basis or origin. Hence the combination of (1) and (2) implies the cones are the physiological basis of unique hues, providing there is no more than one such basis in the visual process. In sum, the cones are indicated or implied (depending on the reader's interpretation of the logic) to be the physiological basis of the unique hues.

Given that the cones are a physiological cause of unique hues, how direct a cause are they? The close correlation suggests the cause is direct rather than cause at a distance. This appears to conflict with the considerable neural interval between receptor layer and cortex where the unique hues are regulated and sensed; the matter is discussed further below.

### Some Other Implications

Although recent research for the neural basis of unique hues focused on LGN and cortex, the present result is hardly a surprise: it supports color vision models' derivation of opponent chromatic responses directly from cone sensitivities, and in particular the model and theory (41) which derives wavelength peaks for *bgy* directly from *SML* cones. As already mentioned, the Standard Model (schema at Fig. 4) has the same general derivations of *bgyr* chromatic responses from *SML* cones as the Eq. (3) model above; that is, *S*≈*b*, *M*≈*g*, *L*≈*y*, and *S*+*L*≈*r*, except for the *L*≈*y* link (see Fig. 4 caption). The early vision model researchers apparently did not recognize the direct correlation between cones and unique hue chromatic responses, due probably to paucity and quality of data on cones at that time.

The result also supports Buchsbaum and Gottschalk's seminal analysis [69] of optimum color information transmission in the retina, which concluded that “opponent type processing in the visual system can be deduced as the next logical step after the three initial cone mechanisms.”

It surprises that principal functions at the start of the visual process (cone responses) are so similar to principal functions at the end (unique hue responses). How did it so evolve? A plausible scenario follows. Before the *M* cone evolved to give humans trichromatic vision, our early vision according to molecular genetics was dichromatic with only *S* and *L* cones [61]. Given the two cones, only two (*b* and y) unique hue chromatic responses existed. The cone response curves, *S* and *L*, directly formed (given the present results) the two unique hue chromatic response curves (*b* and *y* in Fig. 1), whose role was to regulate the unique hue sensation in cortex. There was no need of color mixture. Only two hues existed, unique blue and unique yellow. These nowhere varied toward green or red since no green or red hues existed. As Fig. 1 shows, the two chromatic responses *b* and *y* do not overlap so there cannot be any hue mixture. (If there were, it could only produce desaturation, from complementary colors.) In such a simple system, there is no need for trichromatic or any color mixture, other than varying brightness and saturation of a unique hue to distinguish object colors from their background. Gouras and Zrenner [70] have postulated the origin and functional roles of these two complementary colors in early primate vision. Note that *S* and *L* cones, about 445 and 565 nm, are complementary (white-producing) as are *b* and *y* chromatic response curves and their peaks, and also the unique hues (say, 475 and 575 nm). Such a complementary system is in balance with white/daylight, simplifying chromatic adaptation. This system had no need for intermediate major processes between cone outputs *S* and *L* and the psychophysical chromatic responses *b* and *y*. But as vision evolved to trichromatic color vision the cone outputs and unique hue responses were further separated (e.g., by color mixture). Nevertheless the two sets of functions today remain very similar, as shown above. Despite the cone response functions undergoing subsequent processes, e.g., color mixture, adaptation and induction, it seems they remain recoverable by reverse computation later in the cortex, for unique hue regulation.

The matching of a physiological set of three functions (*SML* responses) with a psychophysical set of three functions (*bgy* responses) seems to be unique in color vision and suggests exceptional importance attaches to these functions. It is not merely a case of two functions matching but two sets of three functions each. The discovery of this match may assist the relating of physiology and psychophysics in color vision (66). In contrast, in auditory science the coincidence between physical (e.g., frequency of auditory stimuli) and psychophysical functions (perceived frequency of sounds) is common.

Given the cone outputs lead directly to opponent-color chromatic responses, where is the physiological evidence for these in retina and LGN? In both areas, spectrally opponent single cells are common and were once thought to represent opponent-colors [9], [71]. But in recent times, these cells are generally agreed to not represent opponent-colors [72], [73] but instead imply complementary colors [41], [74], e.g., colors on opposite sides of the neutral point in a color space. A possible explanation of the missing opponent-color responses lies in a recent theory [41] that opponent-colors, newly produced from the cone outputs, are immediately converted by summation to complementary colors. These, but not opponent-colors, appear frequently in the retinal and LGN data [74], [75], [76].
